# Development of an ostrich-derived single-chain variable fragment (scFv) against PTPRN extracellular domain

**DOI:** 10.1038/s41598-024-53386-5

**Published:** 2024-02-14

**Authors:** Hamed Dabiri, Majid Sadeghizadeh, Vahab Ziaei, Zahra Moghadasi, Ali Maham, Ensiyeh Hajizadeh-Saffar, Mahdi Habibi-Anbouhi

**Affiliations:** 1 Department of Genetics, Faculty of Biological Sciences, Tarbiat Modares University, Tehran, Iran; 2 National Cell Bank of Iran, Pasteur Institute of Iran, Tehran, Iran; 3 Department of Regenerative Medicine, Cell Science Research Center, Royan Institute for Stem Cell Biology and Technology, ACECR, Tehran, Iran; 4 Advanced Therapy Medicinal Product Technology Development Center (ATMP-TDC), Royan Institute for Stem Cell Biology and Technology, ACECR, Tehran, Iran

**Keywords:** Regenerative medicine, Antibody generation

## Abstract

In type 1 diabetes, the immune system destroys pancreatic beta cells in an autoimmune condition. To overcome this disease, a specific monoclonal antibody that binds to pancreatic beta cells could be used for targeted immunotherapy. Protein tyrosine phosphatase receptor N (PTPRN) is one of the important surface antigen candidates. Due to its high sequence homology among mammals, so far, no single-chain monoclonal antibody has been produced against this receptor. In this study, we developed a novel single-chain variable fragment (scFv) against the PTPRN extracellular domain. To this aim, ostrich species was used as a host is far phylogenetically birds from mammals to construct a phage display library for the first time. An ostrich-derived scfv phage display library was prepared and biopanning steps were done to enrich and screen for isolating the best anti-PTPRN binders. An scFv with appropriate affinity and specificity to the PTPRN extracellular domain was selected and characterized by ELISA, western blotting, and flow cytometry. The anti-PTPRN scFv developed in this study could be introduced as an effective tool that can pave the way for the creation of antibody-based targeting systems in cooperation with the detection and therapy of type I diabetes.

## Introduction

Among all diabetes, type I diabetes is an early-onset disease in which the immune system invades and destroys pancreatic beta cells^1^. Type I diabetes patients are insulin-dependent and mostly affect from 4 to 7 and 10 to 14 years. Nevertheless, at the time of diagnosis, about 30–40% of pancreatic beta cells are healthy and able to produce insulin^1^. If the immune system's attacks on the insulin-producing cells stop at the early stages of the disease, it may lead to the prevention of disease progression as the result of beta-cell repair through multiple mechanisms^2^.

The local inhibition of immune system attack, require the use of an antibody as a navigator that target appropriate antigen on the surface of pancreatic beta cells^3^. Selecting the appropriate antigen with distinct criteria is the first step. These criteria include 1. Enough amount of the protein on the surface of pancreatic beta cells 2. Specific RNA/protein expression in pancreatic beta cells. 3. Containing enough and accessible extracellular domain 4. No vital function for cells (which could negatively affect cell function after antibody binding)^4^. One of the most appropriate surface antigens of pancreatic beta cells based on the mentioned criteria is the PTPRN (unpublished data). Production of the appropriate antibody after the selection of the surface antigen is the next step.

The broad family of transmembrane proteins known as receptor-type protein tyrosine phosphatases (RPTP) is engaged in multiple signaling pathways^5^. There are eight different RPTP subtypes. Members of the R8 subtype, ICA512 (also known as PTPRN, PTP35, or PTPRN), include a large ectodomain, one transmembrane segment, and one protein tyrosine phosphatase (PTP) domain that is catalytically deficient. ICA512 is mostly expressed in neuropeptidergic neurons and peptide-secreting endocrine cells, such as insulin-producing pancreatic beta cells^6^. They are found in secretory granules (SGs) and are involved in the development, storage of cargo, transport, exocytosis, and recycling of insulin SGs, and also in cell proliferation. According to sequence information, PTPRN protein has a length of 979 amino acids and is made up of an intracellular domain (amino acid 601–979), a transmembrane segment (amino acid 577–600), and an extracellular domain (amino acid 1–576)^7^. PTPRN's extracellular domain is cleaved in a significant portion during the fusion of insulin-containing vesicles' membrane with the plasma membrane, which leads to the appearance of the 127 amino acid protein on the cell surface^8^. In this regard, the mentioned protein can be considered as a potential target to develop beta cell-specific antibodies.

Antibodies are proteins related to the immune system usually named immunoglobulins. Each antibody contains 4 chains including 2 heavy and 2 light chains. All antibody chains are linked to each other to make a "Y" shaped structure. Among several antibodies, the polypeptide sequence in the head of the "Y" significantly varies. This variable region, composed ordered of 110–130 amino acids, armed the antibody for specific binding to the antigens. The variable region involves the tips of the light and heavy chains. Fragment antigen binding or Fab that contains the variable ends of an antibody can cleave by treating the antibody with a protease. The smallest functional VH-VL domain for binding to antigens is the single-chain fragment variable (scFv), which is around 1/6 the size of the whole antibody^9^. Single-chain fragment variables (molecular weight of 26–28 kDa) that are usually connected by a bendy linker of amino acids are typically created using the phage display technique^9^. Additionally, benefits including reduced immunogenicity, increased tissue penetration capacity, and quicker cleaning from off-target tissues have made scFv an appealing choice for therapeutic purposes^10^. Single-chain fragment variables also can be used in the structure of bispecific antibodies (bsAb), chimeric antigen receptors of T regulatory (CAR-Treg) cells, and labeled antibodies for beta-cell mass (BCM) detection^11–13^. These approaches are useful in both detection and therapy applications for type I diabetic patients.

The construction of antibody phage display libraries is a different method from the conventional hybridoma technology^14^. They contain separate monoclonal antibodies from great immunoglobulin gene collections that are expressed as scFvs on the surface of bacteriophages. According to M13 features, scFvs may be displayed on the surfaces of filamentous phages by introducing a foreign scFv DNA fragment into the gene that codes for the filamentous phage coat protein. Then "panning" or "biopanning," a selection and binding enrichment procedure, is carried out^14^. Dependent on their unique antibody binding ability to the coated antigen, the biopanning permitted the extraction of the best antibody-phage fusions from 10^8^-fold extra phage particles. Therefore, in phage display technology, the mentioned steps lead to more enrichment of the antibody pool and ultimately the selection of antibodies with higher binding ability and specificity against the target antigen^14^. Phage libraries usually generated from animal (or human) variable gene repertoires are constructed from mRNA extracted from B cells of immunized or naïve donors^15,16^. Naïve libraries are used generally in situations that were made from multiple donors but immunized library employment is preferred^15^. Immunization of the host animal by the antigen in multiple steps causes the induction of primary and secondary immune responses leading to the production of more appropriate high-affinity antibodies against target antigen^10^.

Hence, after the selection of an appropriate antigen, choosing a suitable animal as a host for antibody production is another important point. Among the accessible animals, birds, and especially ostrich species have received more attention in recent years for producing different antibodies from mammals^17^. Immunoglobulin Y antibodies (IgYs) from the bird egg yolk have been applied against bacterial and viral infections in humans and animals^18^. Their advantages consist absence of interactions with mammalian Fc receptors, low generation cost, and easy extraction^18^. In comparison to mammalian IgGs, they have higher antigen specificity and impressive binding avidity. They also have been shown notable pathogen-neutralizing activity in multiple diseases^18^. Ostrich species are the best choice for antibody generation against some target antigens^17^. This matter is not only due to the production of a massive bulk of antibodies in the egg yolk but also is mostly due to the phylogenetic distance between humans and ostriches^19^. In other words, human proteins with high homology between mammals do not easily stimulate the immune system of animals such as mice and rabbits to produce antibodies^19^. Instead, ostrich immune cells, due to the difference in sequential and structural motifs that usually exist between human and ostrich antigens, are better stimulated and produce more suitable specific antibodies against the injected antigen^20^. The extracellular domain of PTPRN protein is one of these antigens that has high homology among mammals.

In this study, an ostrich-derived scFv phage library was constructed for isolating a specific anti-PTPRN antibody. Expression of the soluble scFv was carried out in E. coli bacteria and its affinity to PTPRN-expressing cells (Beta-Tc3) was confirmed using characterizing tests such as ELISA, western blotting, and flow cytometry.

## Materials and methods

### Cell culture and ostrich immunization

Beta-Tc3, as a mouse pancreatic beta cell line, was taken from Royan Stem Cell Bank (Royan Institute, Tehran, Iran). The cells were seeded in a high glucose DMEM (Gibco, USA) complete medium which had 10% fetal bovine serum (FBS) (Gibco, USA), and incubated at 37 °C for a week. Upon the cells reached 80% confluency, total RNA extraction using TRIzol reagent (Ambion, UK) was performed from the cells and cDNA synthesis through superscript TM II reverse transcriptase (Invitrogen, MA, USA) with Oligo (dT) primers was done. Consequently, Real-Time PCR was used to validate Beta-Tc3 as PTPRN expressing cell lines. Briefly, two cDNA samples of Beta-Tc3 as a template and GAPDH gene primers as an internal control were used to prepare all PCR reactions, except no template reactions. Triplicate reactions of each PTPRN, SMAD3, and Col1 primer were prepared as test, positive, and negative control genes respectively. The Real-Time PCR was run in an Applied Biosystem (USA) instrument with 40 cycles using Sybergreen reagent (Amplicon, USA).

Finally, two male ostriches, about 8 months old, were vaccinated by Beta-Tc3 cells as immunogen. Around 2 × 10^7^ cells were injected into each ostrich in a subcutaneous and intramuscular administration manner, on day 0. On days 21, 34, and 48, a booster injection containing 1 × 10^7^ cells was administered. One week following the fourth injection, an enzyme-linked immunosorbent assay (ELISA) was carried out to titrate antibodies against nonimmune serum using recombinant human PTPRN antigen (Abcam, USA), at a concentration of 1 µg/ml and serum dilution of 1/1000. Blood was collected from the ostriches according to the standard protocol with ethical considerations three days after the final injection.

The animals who received a standard diet supplemented with Cobaphos multivitamin (Razak, Iran) were kept on a 12:12 h light/dark cycle and regulated room temperature of 22–24 °C during the experiment. The animals were kept for a certain period to enter another project after the healthy condition was achieved. All methods and aspects of the project (Study design, Sample size, Inclusion and exclusion criteria, Randomisation, Blinding, Outcome measures, Statistical methods, Experimental animals, Experimental procedures, and Results) were performed in accordance with the ARRIVE (Animal Research: Reporting of In Vivo Experiments) guidelines and regulations^21^. The experiments on anaesthesia and all animals were also conducted in accordance with the American Veterinary Medical Association (AVMA) Guidelines^22^. All animal procedures were approved by the Royan Institute Ethics Committee (IR.ACECR.ROYAN.REC.1399.030), Tehran, Iran.

### Variable-region amplification of antibody genes

PBMC cells were isolated from ostrich blood by the ficoll gradient density (Lymphodex, Innotrain, USA). Following the manufacturer's recommendations, RNA was extracted from ostrich PBMC cells by TRIzol (Invitrogen, USA). Using superscript™ II reverse transcriptase (Invitrogen, Massachusetts, USA) and Oligo (dT) primers, cDNA was synthesized. The acquired cDNA was utilized for the amplification of the variable light (VL) and variable heavy (VH) segments by PCR using 9 primers (7 primers for VL and a pair for VH) in 13 reactions (1 for VH and 12 for VL) and 28 PCR cycles (30 s at 94 °C, 30 s at 58 °C, and 60 s at 72 °C)^23^. Using the bioinformatic tools, restriction sites of *Not*I and *Mlu*I enzymes were created at the 5′ and 3′ ends of the initial VL amplified products. Like this, the restriction sites of *Nco*I and *Hind*III enzymes were used at the 5′ and 3′ ends of the initial VH amplified products to sever the lateral ends of the products.

### Construction of sub-library and library

According to our previous protocol set up, a sub-library, and phage display library were constructed^24–26^. Briefly, using the GenElute™ Gel Extraction Kit (Sigma, Germany), amplified VL and VH fragments were individually mixed, cut, and extracted from the agarose gel. The purified VL and pSEX81 plasmid (PROGEN, Germany) were cut using the same restriction enzymes, *Not*I and *Mlu*I (Thermo Fisher Scientific, MA, USA), and then ligated into each other. To create a sub-library, the ligated products were electroporated into electrocompetent TOP10 *E.coli*. For this method, the bacteria were plated onto a SOBGA agar. The medium was supplied by ampicillin (100 µg/ml for final concentration) and glucose (100 mM). The plates were put in a 37 °C incubator overnight. An elastic peptide linker (EEGEFSEAR) allowed VH and VL to merge in the pSEX81 phagemid to create the recombinant scFv antibody. For the construction of a complete library, pSEX81 phagemids containing VL segments were isolated from the sub-library using the GenElute™ DNA extraction Kit (Sigma, Germany) and consequently cut with *Nco*I and *Hind*III enzymes (Invitrogen, USA). Similar cutting reactions were done for the pure VH segments and VL-phagemids and ligated by the same concentrations carried out for VL and pSEX81 phagemids. Final ligation products were electroporated into electrocompetent TG1 *E. coli.*

By using colony PCR on randomly chosen clones of TOP10 and TG1 *E. coli* cells which transformed, the ligation efficiency was assessed. Additionally, the size of the library was determined using the number of choices from bacterial colony counts.

### Expression of the extracellular domain of PTPRN protein

In this study, due to the high cost and less amount of commercial antigen, it was decided to produce recombinant antigen to advance the bio-panning steps by using enough PTPRN antigen. For this purpose, the protein sequence of the extracellular domain region of the PTPRN antigen was extracted from the NCBI database. The antigen nucleotide sequence was codon optimized and synthesized. The nucleotide sequence was cloned into the pET15b expression vector (Novagen, Germany). The PTPRN-pET15b construct was extracted and transformed in BL21(DE3) *E. coli* strain (Pasteur Institute, Iran) after multiplication and transformation in Top10 bacteria. Three colonies of the transformed BL21 bacteria were selected to adjust the expression conditions. For this purpose, each clone was grown in 5 ml of Terrific Broth (TB) Medium. After the Optical density (OD) of 0.6 was achieved, Isopropyl β- d-1-thiogalactopyranoside (IPTG) (BioBasic, Canada) in 0.1 mM concentration was employed to induce the expression of the target protein. Samples were taken before, 2 h after, and overnight after induction and consequently analyzed using 12% SDS-PAGE. Finally, the PTPRN antigen was purified with Ni–NTA agarose (QIAGEN, Germany).

### Anti-PTPRN specific binder capture through library screening & selection by biopanning

To rescue the pSEX81 phagemid particles that produce scFv proteins in fusion with the pIII surface protein of M13, M13KO7 helper phage (Stratagene, USA) was used to infect of bacteria library. The MOI of infection was 20. The phage particles were extracted by PEG/NaCl standard protocol in which 2.5 M PEG 6000 in NaCl 20% w/v solution was used. Precipitated phage particles were eluted and titrated for the next biopanning rounds.

100 µl of recombinant human PTPRN protein (50 µg/ml) was introduced to one well of the ELISA plate (Nunc, Denmark) during the first round of biopanning. The phosphate-buffered saline (PBS) (1X) (as the antigen solvent liquid) was then added to the negative control well, and both were kept at 4 °C overnight. The wells coated with antigen and PBS were rinsed four times with PBST, blocked with 1% casein, and then incubated for one hour at room temperature. The process was finished by rinsing with PBS four times. Each well received 100 µl collected phage containing 10^12^ plaque-forming units (PFU) along with 0.05%Tween (PBST). During these steps, scFv antibodies linked to the pIII of phages were attached to the target antigen coated on the bottom of the well. Plate wells were rinsed with PBST five times, after two hours of incubation at room temperature to clear any unspecifically bound phages. Strongly bound phages were separated by employing 100 mM triethylamine (100 µl) treatment for 12 min. They were neutralized with 1 M Tris–HCl (pH 7.5, 100 µl) immediately. Collected phages exposed to the coated antigen were used for infection of TG1 *E. coli* bacteria. The bacteria were applied for the subsequent four panning rounds with growing up and more infection by helper phages.

For more stringent selection, antigen concentrations were reduced to 35, 25, 15, and 10 μg/ml in rounds two to five, separately. Also, the number of rinsing processes was increased to 10-, 15-, 25-, and 30-times during the next rounds.

For the evaluation of single clone screening, 10 µl of extracted phages from each cycle was employed. The biopanning process was accomplished by serially diluting phages to infect TG1 cells, and then cultivating the infected cells on plates of LB agar that included ampicillin antibiotic (100 µg/ml). Anti-M13 antibody (GE Healthcare, USA) conjugated with horseradish peroxidase (HRP) was used to ELISA. The results were illustrated by the absorbance at 450 nm wavelength for 10^9^ extracted phages from each biopanning cycle. The recombinant phage affinities were measured to 1.5 µg/µl recombinant human PTPRN protein.

### Screening through ELISA

Monoclonal phage ELISA was used to screen single clones acquired from bio-panning rounds and choose the most appropriate clone with the desired binding ability to the PTPRN antigen. One hundred and twenty individual clones were tested for the first time on monoclonal phage ELISA. Then eight clones that had significant differences in OD were chosen for repeat monoclonal phage ELISA to confirm the primary ELISA results. After that two individual clones with higher differences between positive (+Ag) and negative (−Ag) OD were regarded for production of soluble scFv proteins and characterization test. For this aim, 120 individual clones were cultured in 2xYT (200 µl) containing ampicillin (100 µg/ml) and glucose (1%) separately. It was then incubated at 37 °C to the exponential growth situation before infection with helper phages around the course of an hour. The bacteria pellets of each clone were soluted in a new 2xYT medium with ampicillin (100 µg/ml) and kanamycin (50 µg/ml). The bacteria were cultured at 28 °C temperature and 200 rpm overnight after being centrifuged to deplete the glucose. For monoclonal phage ELISA, the medium collected from single clone cultures was employed. On Nunc ELISA wells, 1.5 µg/ ml of recombinant human PTPRN protein was coated, and PBS served as the checkpoint. The plate wells were blocked with 1% casein and rinsed with PBST (0.05%) four times. After rinsing, 100 µl of culture medium including phage particles (fused with scFvs) poured into the wells. The plate put two hours at RT.

Five times were spent washing the wells before they were exposed to anti-M13 antibody conjugated to HRP for one hour at RT. These steps were done to identify any phages that had adhered to the coated antigen. After five cycles of washing, the 3,3′,5,5′-tetramethylbenzidine (TMB) (Sigma, Germany) substrate coloring reaction was measured at 450 nm. Approximately 120 distinct clones were examined throughout this procedure.

### Sub-cloning, production & purification of soluble scFv

The nucleotide sequences of the scFv segment in the PSEX81 vector extracted from the 8 selected clones were identified by sequencing (Macrogen, South Korea), and the ALIGN tool (Clustal Omega) was utilized for sequence analysis. To complete the evaluation of the binding ability of scFv to PTPRN antigen, soluble scFv protein was needed. To produce the soluble form of the scFv without fusion with phage pIII, scFv DNA fragments of PSEX81 phagemids for two clones (clone 4 and 5) were cut by *Not*I and *Nco*I enzymes and ligated to the pOP101 plasmid (PROGEN, Germany). The competent *E. coli* HB2151 cells (Pasteur Institute of Iran, Iran) were transformed by the recombinant constructs. 1 mM Isopropyl β- d-1-thiogalactopyranoside (IPTG) was added to the cultured cells in the exponential state, causing the induction of the lac promotor for scFv expression. A signal peptide named pelB included in the pOP101 plasmid subsequently guided the scFv into the periplasmic region. Two buffers were prepared to extract the soluble scFv from the periplasmic space. TES was made of Tris (1 M), EDTA (100 Mm); sucrose (0. 5 M), Ph 8), and TES/4 prepared of 1:3 v/v TES buffer/H2O. The resultant bacteria was soluted in TES and TES/4 buffers and held on ice to start heat shock after osmotic change for three hours for relief of soluble scFv proteins. The incubation process took place at 28 °C and 200 rpm overnight. By a Ni–NTA resin (QIAGEN, Germany) packed column, the positive clone's specific scFv was isolated from the bacteria. Using a 10 kDa Amicon spin column (Merck Millipore, Germany) scFv proteins were concentrated and then evaluated using an ELISA just like the previous stage.

### Specificity & affinity measurement

A competition ELISA using the Pierre Martineau technique was carried out to ascertain the affinity of the anti-PTPRN scFv^27^. Briefly, soluble scFv protein was purified by size exclusion chromatography (Superdex 200 Increase small-scale SEC, Cytiva, USA). Pure scFv protein (2 µg/ml) was added to PTPRN-coated wells to interact with it. Then, different concentrations of free PTPRN antigen (0.39, 0.78, 2, 4, 15, and 40 nM) were added, and the binding capacity was filled out after 1 h of incubation at room temperature. The ELISA was completed as ostrich scFv previous ELISA was carried out. To determine the K_d_ (dissociation constant) as the most important inverse parameter of scFv affinity, the below equation was used^27^.

1 $$A=\left({A}_{max}-{A}_{0}\right)\times \frac{-\left(x-a+{K}_{d}\right)+\sqrt{{\left(x-a+{K}_{d}\right)}^{2}+4{K}_{d}}}{2a}+{A}_{0}$$

In this equation: A was the absorbance, A_max_ and A_0_ were, respectively, the maximal and minimal ELISA signal obtained, K_d_ was the dissociation constant, a was the total scFv concentration, and x was the antigen concentration of A (absorbance).To evaluate the binding ability and cross-reactivity of scFv with cellular PTPRN antigen, western blotting was accomplished with different cell lines. Briefly, the cell lysates of Beta-Tc3, PANC-1, Jurkat, Raji, HepG2, and HUVEC cell lines were run on an SDS-PAGE gel (15%) to identify the expressed PTPRN antigen. The gel was dyed with Coomassie’s brilliant blue R-250. The bands on an identical SDS-PAGE gel were transferred onto a membrane of nitrocellulose. The blotted membrane was first blocked with 1% casein overnight at 4 °C, then incubated for 90 min at room temperature with the anti-PTPRN scFv (10 µg/µl), then 1 h with 1/1000 (v/v) anti-ostrich IgY mouse polyclonal antibody (Pasteur Institute of Iran, Iran). The blot was incubated with 1/2000 (v/v) goat anti-mouse antibody HRP conjugate (Abcam, UK) and finally developed by diethylamino benzidine (DAB) (Sigma, Germany) to check for the presence of protein fragments. The membrane was washed with PBS several times to stop the process. The PBST (PBS with 0.05% Tween 80) was used to wash the membrane after each incubation.

By using flow cytometric analysis, the specificity of the developed scFv in binding to PTPRN protein on the cell surface was also assessed. The anti-PTPRN scFv (10 µg/µl) was first incubated with Beta-Tc3 cells and then with anti-ostrich IgY mouse polyclonal antibody (1/1000 v/v). The final incubation was carried out with 1/1000 (v/v) goat anti-mouse antibody FITC conjugate (Abcam, UK). The flow cytometry instrument (BD FACSCalibur, Germany) was used to measure the intensity of the fluorescent signal, and FlowJo 10 (Treestar, USA) was used to analyze the results.

### Statistics

Statistical analyses were carried out by unpaired t-test method for ELISA results as well as expression level evaluation for Real-Time PCR row data. In ELISA, the OD of scFv-treated wells (antigen coated) was compared with wells that didn’t coat antigens as negative controls. In gene expression analysis of Real-Time PCR, the expression level of PTPRN was compared with Col1 expression as a negative control gene. The samples with *P* Value < 0.1 were considered significant.

## Results

### Analysis of immunization process

**Figure 1:**
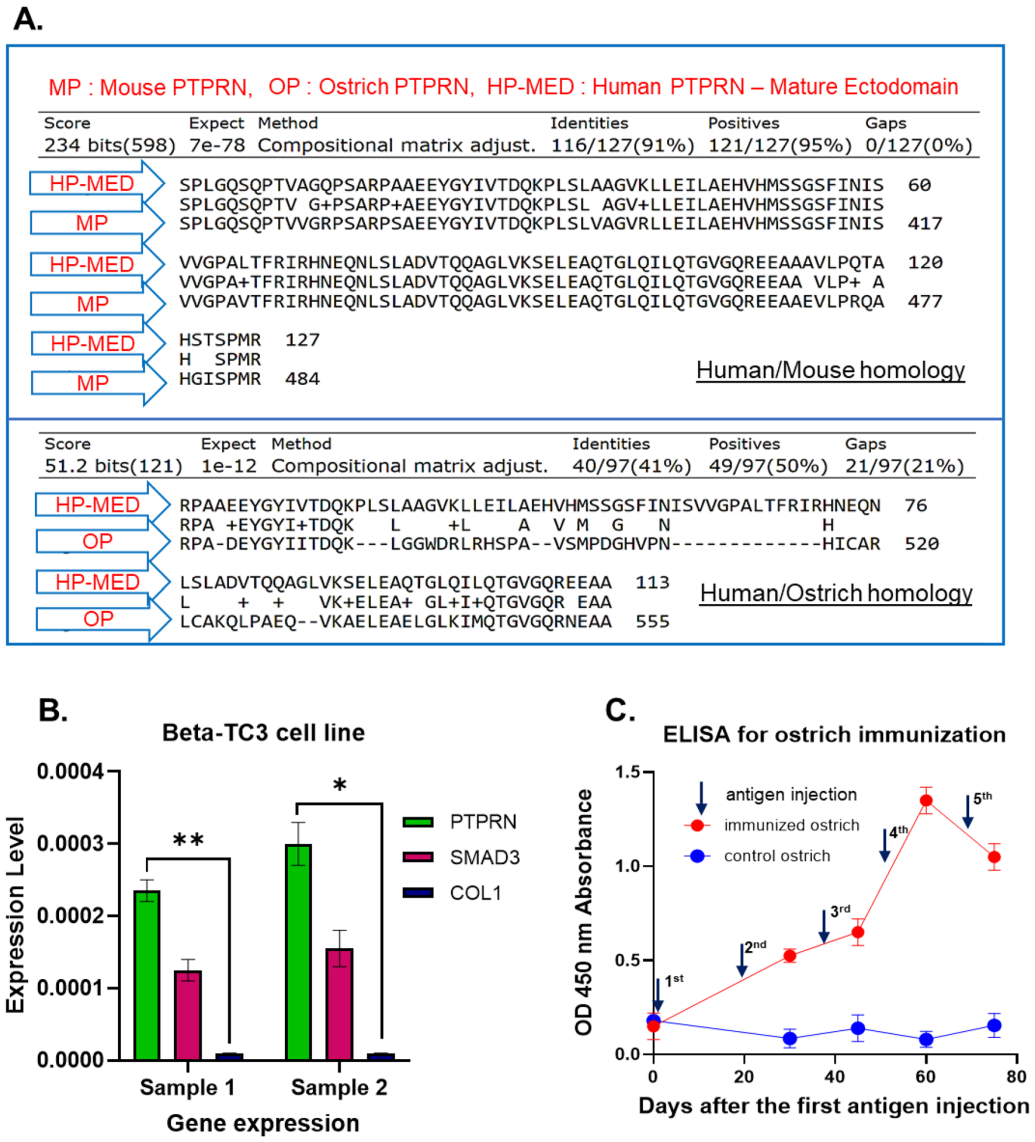
(**A**) Homology of 127 amino acids of PTPRN between the human/mouse and human/ostrich. Less homology (41% identity) was achieved to generate the antibody from the ostrich. (**B**) Real-Time PCR of PTPRN expression in the beta-TC3 cell line. SMAD3 is a positive control gene for beta-cell lines and Col1 is a negative control gene. (**C**) Ostrich immunization ELISA curve. One week after the fourth injection, the highest antibody titer against the human PTPRN was reached. The downward trend appeared after the fifth injection because of the immune tolerance event. ***: *P* Value < 0.001, **: *P* Value < 0.01, *: *P* Value < 0.05, ns; not significant.

Compression of PTPRN extracellular domain sequence of human and several species was achieved by NCBI homology search^28^. The less identity (41%) of 127 amino acids was detected between the human and ostrich sequences (Fig. 1A).

The overexpression pattern of the PTPRN gene on the cytoplasm of Beta-Tc3 cells was found to be acceptable level (Fig. 1B) based on the Real-Time PCR data analysis. The PTPRN gene expression was compared to Col1 as negative control in two samples (*P Value* of samples 1 and 2 were calculated at 0.0044 and 0.0105, respectively). The cultured Beta-Tc3 cells were subsequently employed for ostrich immunization. In comparison to non-immunized serum (collected before antigen injection) and the negative control (non-immunized ostrich), ELISA data confirming immune response in ostrich serum indicated a rising antibody titration trend through injection steps by cell immunization (Fig. 1C). OD450 absorbance of greater than 1 was seen after three antigen injections.

### Construction of scFv phage displays library

**Figure 2:**
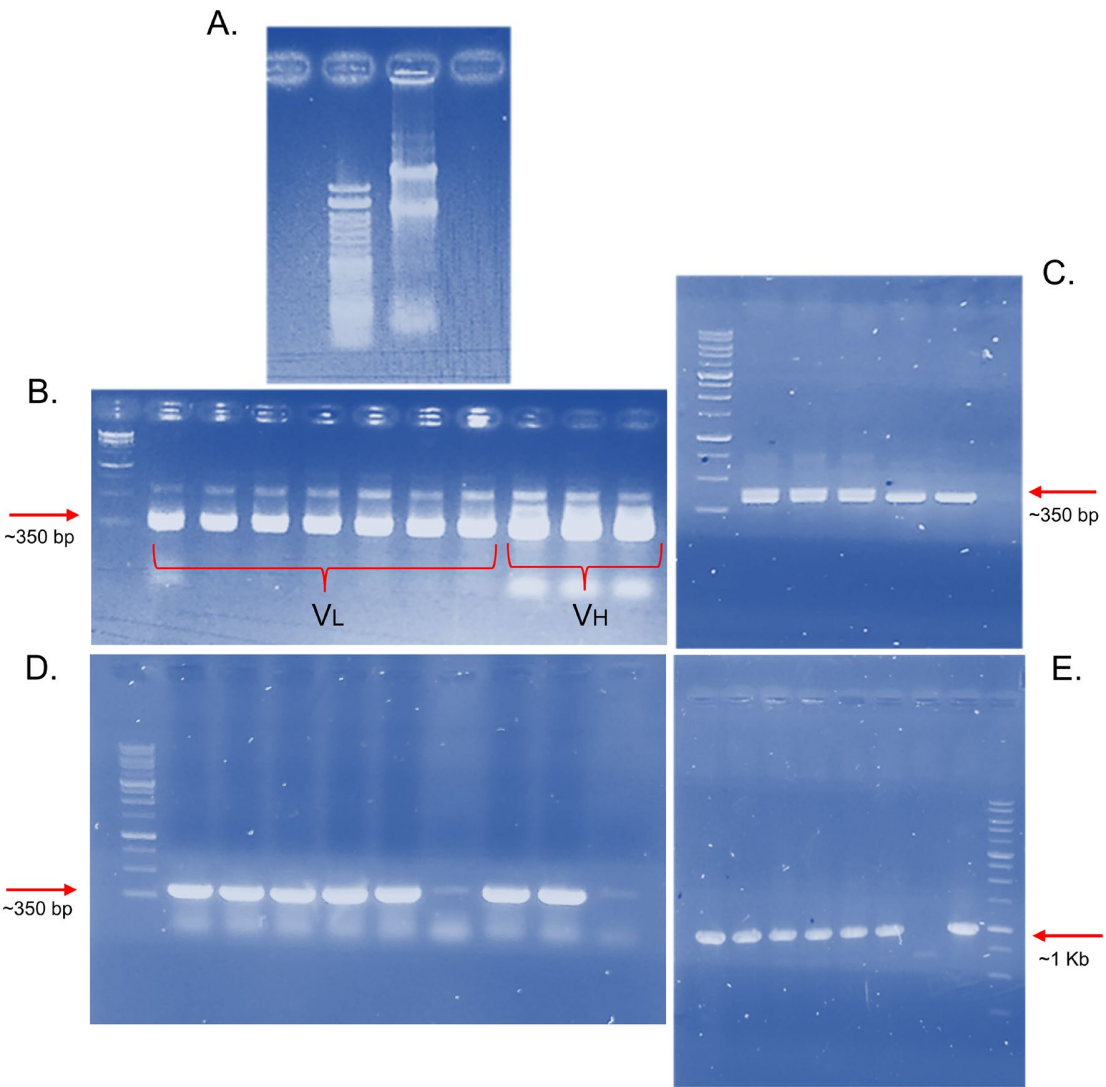
(**A**) RNA extraction from ostrich PBMCs. Two sharp bands with large sizes indicate the true RNA extraction process with less RNA degradation. (**B**) PCR results for amplification of antibody variable fragments (VL/VH) whose RNAs were extracted from ostrich-derived PBMCs. The bands are about 350 nucleotides indicating the true VL and VH amplified products. (**C., D.,** and** E**) illustrating the colony PCR of randomly picked clones from the library. The PCR reactions were done by VL, VH, and phagemid (Forward and reverse) primers respectively for (**C**) (**D**) and (**E**) pictures.

Total RNA was isolated from the immunized ostrich's PBMC cells (Fig. 2A). The entire cDNA was synthesized and VL and VH regions were amplified by PCR to prepare adequate products for cloning. Running an agarose gel (1.5%), the PCR results showed 350 bp bands that corresponded to the VL and VH regions, as was predicted (Fig. 2B). The effectiveness of the sub-library and library was assessed following the two-step cloning of the digested VL and VH sections into the pSEX81 phagemids. Colony PCR was employed to detect VL in the sub-library on 25 randomly chosen clones. Twenty-one out of 25 clones showed 350 nucleotide bands on agarose gel (1.5%), which illustrated an 84% recombination frequency (Fig. 2C).

Insertion of VH fragments into the scFv cassette had been approved by the colony PCR on 25 clones which were randomly picked from the library. As evidenced by the appearance of about 350 nucleotide bands on agarose gel in 24 within 25 clones (Fig. 2D) revealed a 96% frequency of recombination. Finally, counting clones yielded a library size of over 2.1 × 10^8^ CFU (colony-forming units)/ml, showing a suitable efficacy as an ostrich immunoglobulin repertoire. About one kilobase (kb) bands on agarose gel for PCR products by using vector primers indicating the insertion of VH and VL segments into the pSEX81 phagemids (Fig. 2E).

### Expression of the extracellular domain of PTPRN protein in Bl21 bacteria

**Figure 3:**
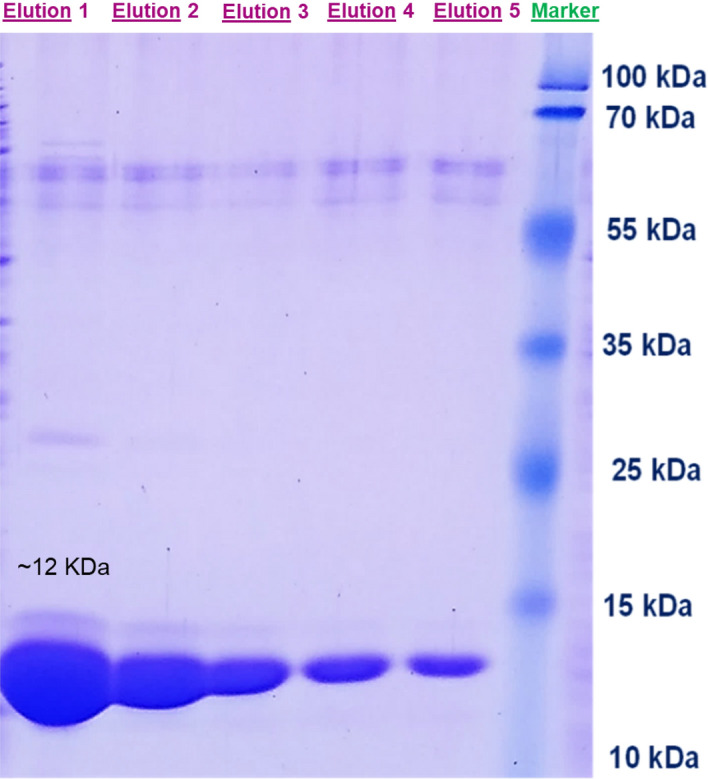
SDS-PAGE image after PTPRN protein purification procedures. Thick protein bands of 12 kDa in eluted samples correspond to the extracellular domain of PTPRN protein which was expressed and purified in *E.coli* bacteria (BL21).

Sharp protein bands of 12 kDa size of the purified samples in SDS-PAGE gel indicated the accuracy of the purification procedures (Fig. 3). Moreover, according to the results, the purity of the extracted antigens is enough and acceptable for the next steps of bio-panning.

### Capture of anti-PTPRN specific binders

**Figure 4:**
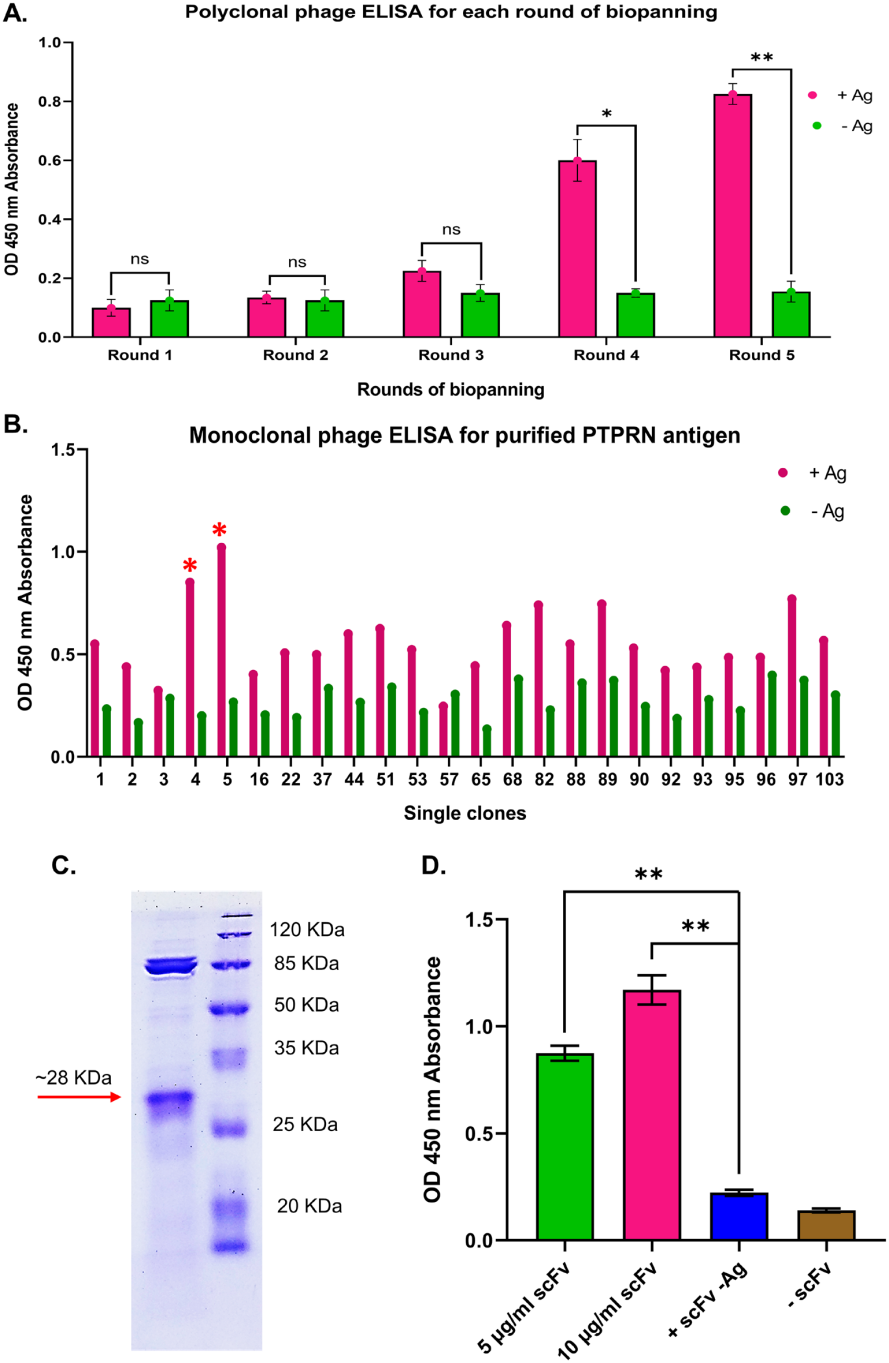
(**A**) Polyclonal phage ELISA after each round of the biopanning process. After four rounds of biopanning, the binding ability of polyclonal captured phages from positive (+Ag) wells reached a significant increase in comparison to negative (−Ag) wells. (**B**) Monoclonal phage ELISA for 120 individual clones to screen specific binders for PTPRN protein. In this graph of Fig. 4, the results of 24 clones that have differences in OD between positive and negative wells have been shown. Hence, two individual colones (four and five) rescued phage particles that displayed scFvs which showed OD of more than a two-fold difference between the positive (+Ag) and negative (−Ag) wells. (**C**) The scFv protein of clone 5 was purified from the HB2151 bacteria by Ni–NTA resin. The band of about 28 kDa in the SDS-PAGE image corresponds to the soluble scFv protein from clone 5. (**D**) ELISA with 5 and 10 µg/ml concentration of soluble scFv which was obtained from clone 5 phagemid. Significant increased OD in ELISA with soluble scFv from clone 5 was reached in 5 and 10 µg/ml concentrations of scFv proteins compared to negative controls (+Ag/-scFv and −Ag/ + scFv) (***: *P* Value < 0.001, **: *P* Value < 0.01, *: *P* Value < 0.05, ns; not significant).

To specifically capture binders for the anti-PTPRN antibody, produced recombinant phages having scFvs with various affinities to target antigen were exposed to coated antigen in 5 rounds bio-panning process. The results illustrated that eluted phages had a considerable rise in titration due to enrichment from 10^5^ PFU in the first cycle of panning to 1.3 × 10^8^ PFU in the 5th cycle. In each cycle, this rise in phage production was also seen when compared to the quantity of phage that was obtained from non-antigen coated wells (including 1 × 10^5^, 3 × 10^5^, 1 × 10^6^, 2.1 × 10^7^, and 1.3 × 10^8^ PFU from the first to the 5th rounds, respectively) (Table 1). These results demonstrated that the selection procedures of specific binding clones and enrichment during biopanning were correctly completed. To evaluate the existence of a suitable enrichment of the bio-panning process, polyclonal phage ELISA was also carried out. According to the results of polyclonal phage ELISA, in comparison to the negative control wells (no antigen coated), the specific binding of obtained phages to target antigen has gradually increased in individual two late cycles (Fig. 4A). OD of polyclonal ELISA for the cycles of biopanning was reached from 0.1 for the first round to 0.6 and 0.8 for the 4th and 5th rounds, respectively (Fig. 4A). The results showed significant differences in OD between positive (+Ag) and negative (−Ag) wells for rounds four (*p* Value = 0.0126) and five (*p* Value = 0.0028).

**Table 1:** Enrichment evaluation of five rounds of the bio-panning process with entered and yielded phages from the library.

Rounds	Phage applied	Phage eluted (+)	Phage eluted (−)	^†^Elution factor
1	10^12^	1 × 10^5^	2.3 × 10^4^	4.3
2	10^12^	3 × 10^5^	4 × 10^4^	7.5
3	10^12^	1 × 10^6^	6 × 10^4^	16.6
4	10^12^	2.1 × 10^7^	5 × 10^4^	420
5	10^12^	1.3 × 10^8^	5 × 10^4^	2600

^†^Elution factor = extracted phages from PTPRN-coated well/extracted phages from non-antigen-coated well.

Monoclonal phage ELISA was carried out with 120 individual clones to find the most suitable single clones (Fig. 4B). Monoclonal phage ELISA was repeated for eight clones with significant differences between positive (+Ag) and negative (−Ag) to confirm the results of the primary ELISA (Supplementary File1). According to monoclonal ELISA findings, among all clones, clones 4 and 5 had greater binding ability to PTPRN in comparison to the negative control since the OD difference reached 0.6 and 0.8, respectively. OD of other clones was equal or less than two-fold in comparison to negative wells. Therefore, only clones 4 and 5 were chosen for subsequent evaluations and characterizations.

Hence, they were sub-cloned into the pOPE101 vector. By sequencing, The results showed that the nucleotide sequence of 8 scFvs from individual clones (including clones 4 and 5) was matched to the worldwide ostrich immunoglobulin sequences from the NCBI database (data not shown). The results revealed that 8 scFv sequences were identical in framework regions (FR-1,-2,-3, and-4) and also were identical to ostrich immunoglobulin sequences deposited in NCBI databases. The result also illustrated that the Hypervariable region, or complementary determining region (CDR-1,-2, and -3) of scFv sequences of two clones (clones number 1 and 51) were identical. In comparison, the 6 remaining clones (clones number 4, 5, 53, 82, 89, and 97) had different CDR sequences from each other and also were different from the ostrich immunoglobulin sequences from the NCBI database.

According to the SDS-PAGE results, the purified scFv protein appeared as a ~ 28 kDa-band which confirmed the correct size of the expected scFv protein of clone 5 (Fig. 4C). The conserved binding ability to PTPRN antigen was observed from the soluble scFv of clone 5. This finding was supported by the 450 nm absorbance level of the purified soluble scFv of clone 5, which was chosen for further investigation (Fig. 4D). A non-significant variation was observed between the results of ELISA for soluble scFv with different concentrations (5 µg/ml and 10 µg/ml). The results showed that the OD of ELISA for soluble scFv was significantly increased in comparison to negative controls (Fig. 4D). The results revealed that the soluble scFv of clone 4 can not bind to the antigen (PTPRN) by ELISA. This may be due to the conformation change of the scFv protein which was separated from the pIII phage protein (data not shown).

### Characterization of the candidate anti-PTPRN scFv

**Figure 5:**
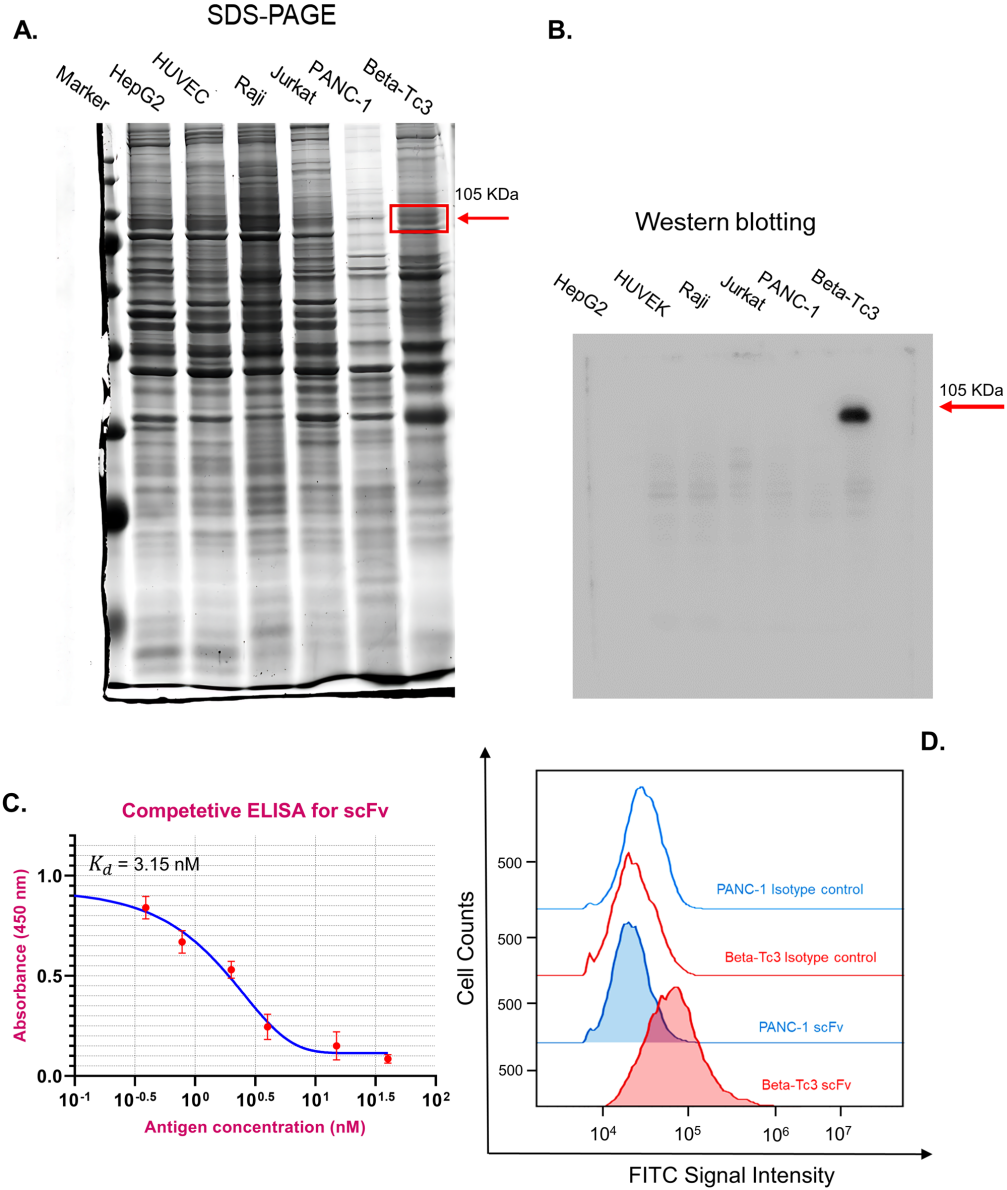
(**A**) SDS-PAGE image of PTPRN positive (Beta-Tc3) and negative () cell lines. Total proteins were extracted from the candidate cells and were loaded into separate wells of SDS-PAGE gel and electrophoresed. A band of about 105 kDa corresponding to PTPRN protein is visible in Beta-Tc3 cells but not in others. (**B**) Western blotting image for cross-reactivity analysis using the ostrich candidate scFv. A band of about 105 kDa related to PTPRN protein was obtained in Beta-Tc3 cells but not in others. Considering that a significant band is not observed in other cells, it can be illustrated that the scFv antibody has less cross-reactivity with the proteins of the negative control cells. (**C**) Affinity diagram of the scFv binding to PTPRN antigen. The results showed that upon the free antigen concentration increased, the amount of free antibody for the next step of binding to the coated antigen decreased, and consequently the OD in ELISA declined. So, the negative sigmoidal regression of the graph was observed. K_d_ was determined at 3.15 nM. K_d_ is the antigen concentration in which half of the scFvs are bound and half of them are free. With this definition, in addition to the number obtained from the formula, K_d_ can also be determined from the graph. (**D**) Flow cytometry was done to evaluate the specificity of scFv binding to cell surface PTPRN antigen. According to the results, the FITC signal for Beta-Tc3, PANC-1 cells, and the control isotypes showed the specific binding of scFv to the surface of most of the Beta-Tc3 but not PANC-1 cells or isotype controls.

To confirm the binding ability of candidate scFv (clone 5) to target antigen (PTPRN), western blotting, flow cytometry, and affinity evaluation by ELISA were employed. For western blotting, primarily, the total protein of PTPRN positive (Beta-Tc3) and negative (PANC-1) cells was extracted and run on a 15% SDS-PAGE gel. The results showed that on the Coomassie-stained gel, a protein band around 105 kDa could be observed which corresponds to the pancreatic isoform of the PTPRN protein. The PANC-1 cell line utilized as the negative control did not exhibit any bands in this size (Fig. 5A and Supplementary 2). To evaluate the affinity of the scFv to the cellular form of the PTPRN a western blot analysis was done with soluble scFv. The results illustrated that the scFv can bind to PTPRN (105 kDa) in western blotting. Moreover, the results revealed that in comparison to other cell line lysates such as Jurkat, Raji, HUVEC, and HepG2, candidate scFv had a specific binding to PTPRN antigen with less cross-reactivity to other proteins expressed in the mentioned cell lines (Fig. 5B and Supplementary 2).

To measure the affinity of scFv, the competitive ELISA was used. By the Pierre Martineau technique, based on Eq. 1, the results revealed that the dissociation constant (K_d_) of scFv against PTPRN was determined about at 3.15 × 10^–9^ M, showing an appropriate affinity in soluble form (Fig. 5C)^27^. K_d_ is the most important parameter that reversely corresponds to the affinity of the antibody. Another definition of K_d_ is the concentration of antigen in which half of the antibodies are free and half of the antibodies are bound.

Additionally, flow cytometry was employed to identify the precise binding of the scFv to the cell surface PTPRN protein in natural form. The results showed that the scFv had specifically bound to the Beta-Tc3 cell lines, which express PTPRN proteins, and located them into the plasma membrane as well as the cell surface. The PANC-1 cell lines, as the negative controls, did not have any additional binding of the scFv in comparison to isotype controls which didn’t incubate with the scFv (Fig. 5D).

## Discussion

Over 400 million people worldwide suffer from diabetes, a metabolic disease that is one of the major causes of death, particularly in undeveloped countries^29^. Today, many kids suffer from type 1 diabetes and scientists have always been looking for a solution to improve the patient's condition. In type 1 diabetes disease, the immune system of the patient attacks and destroys beta cells which are insulin-producing^30^. To prevent immune system attacks, several strategies have been examined till now. Strategies that suppress the immune system generally or locally. In recent years, the superiority of local suppression scenarios has been proven. Therefore, the importance of targeting the beta cell surface proteins becomes more clear for local suppression of the immune system in type I diabetes^31^. For this aim, the development and usage of specific antibodies against these proteins is introduced as the best way^32^. Antibodies that bind specifically with high binding ability to the beta cell surface proteins^32^. Among the multiple ways to produce the novel antibody, the phage display technique using the immunized library for the target antigen is the best procedure to reach the high-affinity single-chain antibody fragments (scFv) that we used in this study^14^.

Interesting applications for beta-specific antibodies are conceivable. The applications include the production and usage of bispecific antibodies (bsAb), CAR Treg cells, and labeled antibodies for monitoring the beta-cell mass (BCM) in type 1 diabetes patients^33^.

Immunoglobulin Y (IgY) refers to antibodies that are produced in the body of birds and can be extracted from egg yolk^18^. Today, IgY has many applications in diagnosis and treatment, especially in infectious and viral diseases^34^. IgY has many advantages over the other types of antibodies^34^. Among all, we can mention more stability as a result of increasing disulfide bonds in the structure, not inducing the mammalian complement system due to the absence of Fc, and easy access as a result of extraction from the egg yolk^34^. In addition, due to the phylogenetic distance between birds, especially ostriches, and humans, stimulating their immune system with human proteins for the generation of the best antibodies is another advantage of using avian antibodies (IgY) for diagnosis and treatment^35^.

Antibodies that are bispecific (bsAbs) have two antigen-binding sites^36^. They are composed of two distinct fragment crystallizable (Fc) regions or two antibodies linked by a common fragment antigen-binding (Fab) arm^36^. Beta-specific antibodies can be used to improve type I diabetes in this way like other autoimmune diseases. Moreover, some efforts have been made to produce CAR-T reg cells for Type I diabetes such as anti-insulin and anti-Hpi CAR Treg cells do not have enough satisfied results^37^. Therefore, the result of the present study, the development of scFv against PTPRN as pancreatic beta cells, could be used for the generation of CAR-T reg cells for type I diabetes in future works.

The metabolic consequences of the type I diabetes, such as glucose level, glycated hemoglobin (HbA1c), insulin or C-peptide levels, which are indications of beta-cell activity, are measured by current diagnostic procedures^38^.

The level of blood glucose, the amount of blood glycated hemoglobin (HbA1c), and also the blood level of the insulin hormone or C-peptide are such as metabolic consequences of type I diabetes which are indications of pancreatic beta cell activity and are measured by current diagnostic procedures. However, they give inadequate information and represent the disease development incorrectly^38^. Since beta-cell mass (BMC) acts directly in the physiopathology of diabetes, it has been proposed as an alternate approach to monitor the progression of type I diabetes in accompaniment to insulin-producing cell activity^39^. However, the study of the BMC imposes very invasive and probably dangerous actions, such as taking biopsies from pancreatic tissues, which makes the condition difficult to diagnose and monitor^39^. The use of radiation-emitting tracers in the field of nuclear medical imaging technology has been recommended as an effective non-invasive manner for the BMC^40^. Positron emission tomography, which is a sensitive and high-resolution platform, offers a supreme strategy for the imagining of BMC, which is mainly important to assess the disease progression and for estimating the effects of treatment on the renewal of the BMC^40^. Thus, the creation of innovative, proven antigens targeted at BMC imaging is crucial and would result in significant advancements in the study of diabetes and its therapies^40^. However, the area of BCM imaging has made significant strides recently, as seen by the several tracers that have been investigated in these studies, and have a rising prospect of making it to human clinical trials^41,42^.

Success in the production of pancreatic beta cell-specific monoclonal antibodies largely depends on choosing the right antigen. Therefore, selecting the appropriate antigen with exact criteria is one of the preliminary steps. High-level protein expression in pancreatic beta cells, specifically expression in pancreatic beta cells, having enough and accessible extracellular domain, and having no vital function for cells are some important criteria in this regard. Based on our comprehensive bioinformatic investigations, one of the most appropriate surface antigens of pancreatic beta cells based on the mentioned criteria is the PTPRN protein (unpublished data). PTPRN protein is one of the surface proteins of insulin-producing cells in pancreatic islets. Upon the fusion of the vesicle membrane with the plasma membrane, the PTPRN protein appears on the cell surface^43^. PTPRN is made up of four different domains: a signal peptide (amino acids 1–24), extracellular (amino acids 25–576), transmembrane (amino acids 577–600), and intracellular (amino acids 601–979)^44^. Several studies have been conducted in relation to the structure and function of this protein^43^. In 2011, Mr. T Cai et al. showed mice that had been knocked out for the PTPRN gene had several physiological problems, including a significant reduction in dense core vesicles of insulin and insulin secretion^45^. Autoantibodies against PTPRN protein are used to identify the onset of the disease in people suspected of type I diabetes^46^. Interestingly, the PTPRN protein does not have a vital function for the normal cell when it is located on the cell surface^47^. In fact, after performing its functional activity among insulin secretion, it can be targeted by antibodies. This protein has three dominant isoforms that are expressed in beta cells of pancreatic tissue as well as the nervous system. Considering the role of transport of neurotransmitter-containing vesicles to the axon terminal in the nervous system, as well as the role of insulin-containing vesicles in beta cells, the protein exposed place in the pancreas is the cell surface and in the nervous system is in the angstrom space of the synapses^45^.

In recent years, various antigens have been used to target pancreatic cells in type I diabetes, antigens such as insulin protein, CUZD1, ZNT8, GLUT1, etc^48,49^. This targeting is done using monoclonal antibodies, recombinant antibodies (bispecific), and CAR Treg cells^49^. The products in question have been produced to prevent the immune system from attacking pancreatic beta cells or to identify the number of healthy beta cells in type I diabetes patients^49,50^. We attempted to use our knowledge for targeting pancreatic beta cells in the current investigation by producing an anti-PTPRN scFv with the appropriate affinity.

The initial stage in this process was to collect antibody genes from animals who had received vaccinations. We investigated cell lines with RNA/protein expression of PTPRN as suitable substitutes for the target protein to produce a possibly efficient antibody. A novel technique that uses an antigen expression cell line as the immunogen is whole-cell immunization^51^. This approach, which differs from conventional immunization methods in that it avoids complicated purification procedures and benefits from naive antigen conformation, is particularly well suited for the identification of new antibodies against membrane protein (extracellular domain)^51^. Increased immunogenicity, unrestricted conformation and alteration of the target of interest getting beyond the difficult purification stages, and one of the finest techniques for finding anti-membrane protein antibodies are some of the featured benefits of whole-cell immunization^51^. The amount of immunological response to the cell lines in two ostriches revealed the presence of antibodies with appropriate specificity, maturation, and affinity against the immunogen, as was expected.

It was important to use primer pools that could cover the diversity of genes to build an effective gene collection for the phage display library to reach a reasonable size. Therefore, we aligned all immunoglobulin transcripts of ostrich deposited in the NCBI database. The VH and VL segments that were amplified demonstrated the effectiveness of the optimized and applied primers that ultimately led to the achievement of adequate recombination rates in the sub-library and library. The results confirmed the availability of a significant number of recombinant clones for further examination. It has been highlighted, however, that the subject of library size usually is inversely correlated with the affinity of the obtained scFvs. Using conventional methods, we were able to produce an ostrich scFv library with 3.1 × 10^8^ CFU/ml, which is consistent with the library size of the phage display technique, as published^24^.

Scientists have shown that fine enrichment preservation typically leads to positive clones in biopanning and true phage library screening to capture specific binders (scFvs)^52^. They also have expressed weak library-enriched repertoires, which do not significantly bind phages to the potential targets^52^.

A binding strength between an antibody and a simple hapten or antigen is defined as ‘antibody affinity’^27^. The affinity of a certain antibody can be measured using different manners. One of the useful accessible ways is competitive ELISA that can be carried out for each novel antibody^27^. By the results of the competitive ELISA, the negative regression curve can be prepared that be used to determine the dissociation constant (K_d_). K_d_ is the most important parameter that inversely corresponds to the affinity and is usually regarded as the measure of antibody affinity. In fact, the smaller the K_d_ number, the higher the affinity of the antibody, and vice versa^27^. In this study, we calculated the K_d_ of obtained scFv by Eq. 1, 3.15 × 10^9^ M which was appropriate. The range of K_d_ for applicable antibodies is usually between 10^6^ M for relatively low affinity to 10^12^ M for very high-affinity antibodies^53^.

Our findings showed that the quantity of extracted phages increased after each panning cycle, confirming the reasonable value of biopanning, and enabling a successful clonal collection with the appropriate affinity^52^. Three exemplary positive clones were produced following five rounds of selection in a work by Wang et al. In this research, an anti-Cry1C scFv was isolated from clone H6 of the human scFv library which was chosen for more characterization^54^. Positive clones were discovered in a different study by Makvandi Nejad et al. In this study, an immunized-derived phage display library was constructed for screening to capture the best anti-monensin antibiotic scFvs^55^. The library size of the two mentioned investigations was 1.47 * 10^8^ and 1.37 * 10^8^ CFU/ml, respectively. The number is close to the library size of our present study (3.1 × 10^8^ CFU/ml)^56^. In 2020, Schoenenwald et al. developed four chicken-derived anti-Usutu virus scFvs^57^. Moreover, Ge et al. produced IgY-derived scFv from chicken species that target the Canine Parvovirus capsid protein^58^. In both investigations, about 100 clones were selected for the first time during the screening procedure to capture the best binders^57,58^. The number of clones is close to our number of screening clones (120 clones). The library size of Schoenenwald et al. was 1.5 * 10^6^ CFU/ml and the library size of Ge et al. was 8.2 × 10^11^ pfu (plaque-forming units)/mL. In both studies, they can characterize the obtained scFvs by western blotting^57,58^. In our study, we can characterize the obtained scFv by western blotting as well as flow cytometry.

In our study, the scFv of clone five detected PTPRN antigen on the surface of the pancreatic beta-cell line. Similar findings have been reported in studies evaluating the scFv binding to biomarkers of several cancers, revealing that such recombinant monoclonal antibodies (Mabs) can be reflected as detection tools to pave the road for future use in cancer diagnosis and therapy.

Significant advances will be made in the use of monoclonal antibodies derived from phage display, especially scFv, to the extent that certain scFvs are currently undergoing clinical trials. In fact, the use of scFv has some disadvantages, such as its short-term persistence at the target tissue due to fast removal from the blood circulation by the kidneys. This poor stability can be almost resolved by conjugating them with different types of proteins such as suppressive cytokines or using them in the structure of CAR-Treg cells to target the desired location. Based on the Serge protocol we characterized a scFv antibody from one individual clone (number 5) that had a higher binding ability to antigen by ELISA, but maybe be other 7 selected clones are appropriate likewise for some applications such as the production of CAR-Treg cells^59^.Besides the growing advantages of using the ostrich species as the host animal for the generation of the scFvs against specific human targets and using them in the therapeutic approach, there are some disadvantages to that^60^. For example, in spite of ostriches producing huge amounts of antibodies and having a good number of lymphocyte cells for phage display library preparation, in the first step of scFv production, keeping ostriches is a rather difficult task. On the other hand, despite the scFv protein with a size of about 30 kDa usually doesn’t induce the immune system, induction of human immune cells against a therapeutic ostrich-derived scFv is not zero. Therefore, in some cases that will have immunological side effects, the humanization step of the applicable scFv should be added to reduce the side effects and increase their half-life in the blood^60^.

## Conclusions

PTPRN antigen appears on the surface of beta cells after insulin secretion and has been assessed as a practical tool to target pancreatic beta cells. In this study, corresponded scFv with appropriate affinity to beta cells was efficiently selected for the first time and its binding to natural PTPRN antigen was illustrated by ELISA, flow cytometry, and western blotting techniques. It can be suggested that PTPRN scFv could be considered a promising tool for different diagnostic and therapeutic applications for diabetic patients.

### Supplementary Information

Supplementary Information 1.

Supplementary Information 2.

## Data Availability

The datasets generated during the current study are available in the ENA (European Nucleotide Archive) repository (Accession Number: PRJEB67560) or web link (https://www.ebi.ac.uk/ena/browser/view/PRJEB67560). Also, the required data are available from the corresponding author (Dr. Ensiyeh Hajizadeh-Saffar) upon request.
