# Supplementary figures and images for: Development of an ostrich-derived single-chain variable fragment (scFv) against PTPRN extracellular domain
Source: Sci Rep. 2024 Feb 14;14:3689. doi: 10.1038/s41598-024-53386-5 (PMC10866909; doi:10.1038/s41598-024-53386-5)

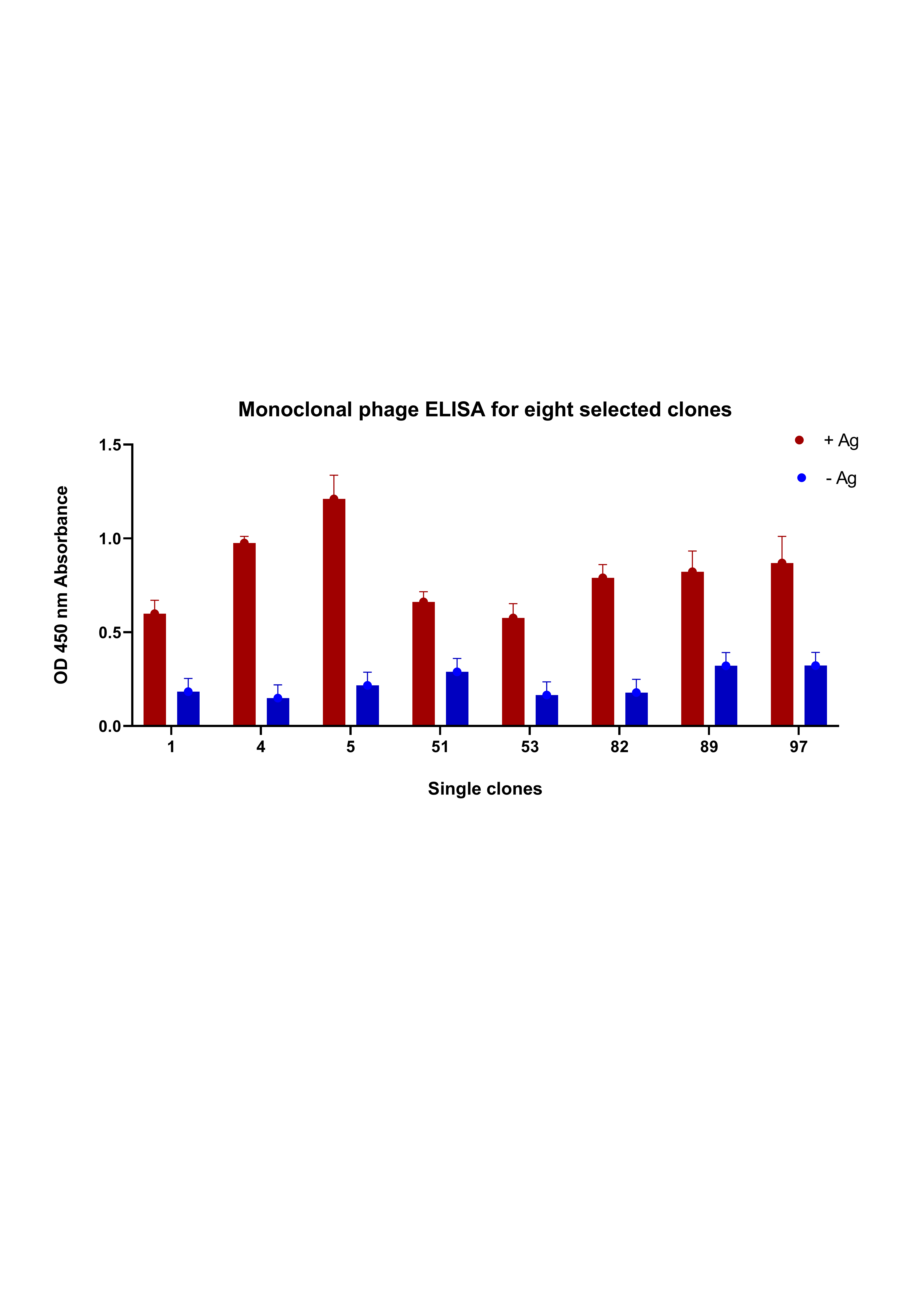

Supplement: Supplementary file 1 — Supplementary Information 1. [file 41598_2024_53386_MOESM1_ESM.png]

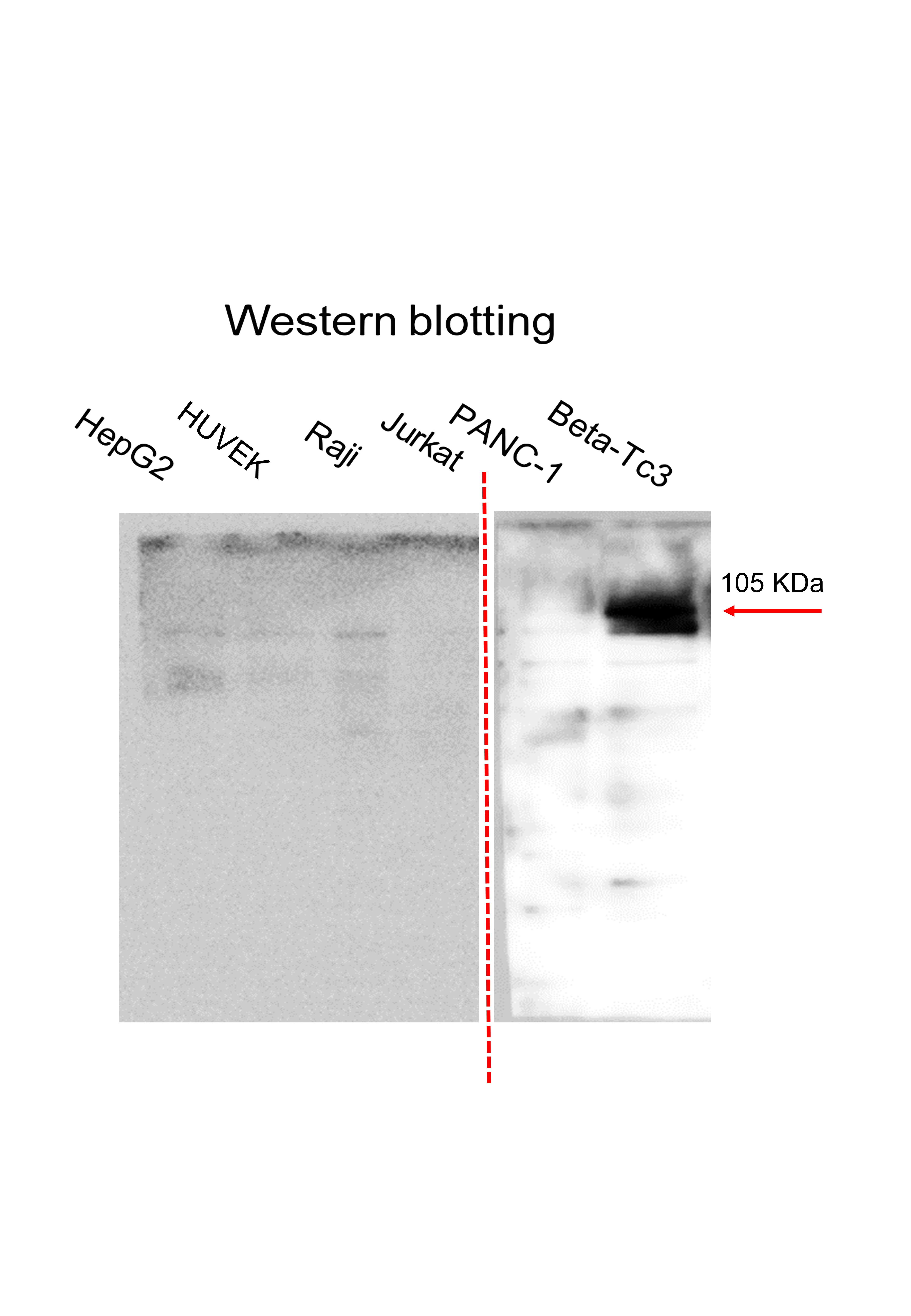

Supplement: Supplementary file 2 — Supplementary Information 2. [file 41598_2024_53386_MOESM2_ESM.png]
